# Treatment of urinary incontinence in women with chronic obstructive pulmonary disease—a randomised controlled study

**DOI:** 10.1186/s13063-021-05816-2

**Published:** 2021-12-11

**Authors:** Stacey Haukeland-Parker, Bente Frisk, Martijn A. Spruit, Signe Nilssen Stafne, Hege Hølmo Johannessen

**Affiliations:** 1 Department of Physical Medicine and Rehabilitation, Østfold Hospital Trust, Grålum, Norway; 2 Department of Health and Functioning, Western Norway University of Applied Sciences, Bergen, Norway; 3 Department of Research and Development, CIRO+, Horn, The Netherlands; 4 Department of Respiratory Medicine, Maastricht University Medical Center (MUMC+), Maastricht, The Netherlands; 5 Faculty of Health, Medicine and Life Sciences, NUTRIM School of Nutrition and Translational Research in Metabolism, Maastricht, The Netherlands; 6 Department of Public Health and Nursing, NTNU Faculty of Medicine and Health Sciences, Trondheim, Norway; 7 Clinical Services, St. Olavs Hospital, Trondheim University Hospital, Trondheim, Norway; 8 Faculty of Nursing, health and laboratory science, Østfold University College, Fredrikstad, Norway

**Keywords:** Chronic obstructive pulmonary disease, Urinary incontinence, Pelvic floor muscle training, Cough-suppression therapy

## Abstract

**Background:**

Little is known regarding treatment of urinary incontinence (UI) in women with chronic obstructive pulmonary disease (COPD). The aim of the study was to explore the efficacy of pelvic floor muscle training (PFMT) or cough-suppression techniques (CST) on UI in women with COPD.

**Methods:**

A three-armed, two-centred, single-blinded, randomised controlled study was performed. Subjects were randomised to (a) PFMT for 16 weeks, (b) 2–3 educational sessions in CST, or (c) written information only. All participants completed questionnaires about UI, cough symptoms, and health status and underwent clinical examinations to evaluate the strength of the pelvic floor muscles and exercise capacity. Daily physical activity levels were measured using an activity monitor and lung function with spirometry. With a significance level of 5% and an 80% chance of detecting a significant difference between groups of 2.5 points on the ICIQ UI SF score, our sample size calculation showed that a total of 78 women, 26 in each group, was required to complete the study.

**Results:**

During the period 2016 to 2018, 95 women were invited to the study. A total of 42 were recruited, three were excluded and 10 (24%) dropped out during the follow-up period. Mean ICIQ-UI SF total baseline score was 9.6 (range: 1–17) and 7.0 (range: 0–16) at follow-up. Changes in subjective UI as measured with the ICIQ-UI SF questionnaire were seen in the PFMT group and control group, but not in the CST group.

**Conclusion:**

Due to the low number of available participants and recruitment difficulties including practical issues such as travel distance, lack of interest, poor state of health, and high number of comorbidities, our results are inconclusive. However, reduced subjective UI was observed in the PFMT and control groups with a trend towards best effect in the PFMT group. Screening for UI is advisable in all women with COPD to be able to identify and treat these women to reduce symptom burden and improve quality of life. Future studies should focus on barriers to recruitment as well as randomised controlled studies with larger sample sizes.

**Trial registration:**

ClinicalTrials.gov NCT02614105. 25th November 2015.

## Background

Urinary incontinence (UI) is any involuntary loss of urine. Prevalence increases with age and women are more exposed than men due to pregnancy, childbirth and menopause [1–4]. Stress urinary incontinence (SUI) is associated with activities involving increased intra-abdominal pressure such as physical exertion, sneezing, coughing or laughing [5]. Respiratory disease is considered a risk factor for UI due to coughing and dyspnoea [6] which increase intra-abdominal pressure increasing stress on the bladder. This may in turn lead to leakage if the pelvic floor muscles (PFM) are weak. In addition, regular coughing may further weaken ligaments in the pelvic floor resulting in UI [7]. Dyspnoea and chronic cough are common symptoms in patients with chronic obstructive pulmonary disease (COPD) [8] and a significant number of women with COPD also suffer from UI [6, 9–13]. Prevalence of SUI in the general population of women is reported to be between 25 and 40%, whereas approximately 50% of women with COPD suffer from UI with SUI being the most common form, presumably due to coughing [9]. UI is associated with lower health-related quality of life (HRQL) in people with COPD, especially amongst women [14].

Although UI is not a life-threatening condition, it is common and can have numerous negative psychological, social, and economical effects. Women with UI in the general population have higher levels of depression, anxiety and stress, poor sleep quality and significantly lower HRQL [15–17]. Additionally, long term UI may result in absence from work, increased healthcare costs, social isolation and physical inactivity [15, 17, 18]. Inactivity is common from the early stages of the COPD due to dyspnoea [19, 20]. Furthermore, inactivity is associated with risk of hospital admissions [21] and is a strong predictor for mortality [22]. The combination of UI and COPD may therefore result in a double risk factor in terms of exercise avoidance and the subsequent negative consequences of inactivity.

There is currently poor evidence regarding the population of women with COPD who develop UI and the impact UI has on their lives. In addition, the current available evidence regarding treatment options for women with COPD and coexisting UI is scarce. Pelvic floor muscle training (PFMT) is well documented for reducing SUI in other populations [4], but only one small study has explored the effect of PFMT in a COPD population. However, the study included few participants, no controls, and PFMT was given in combination with other interventions [23]. Cough-suppression therapy (CST), which involves a range of physiotherapy techniques to focus on identifying triggers and utilising techniques to suppress unnecessary, unproductive coughing, has been demonstrated to positively benefit patients with refractory cough in terms of symptoms and HRQL [24, 25]. It is therefore considered that CST may potentially be effective in reducing cough and subsequently SUI, however, there is no current evidence for CST amongst people with COPD and UI. Therefore, the aim of this study was to explore the effect of PFMT and CST in reducing UI amongst women with COPD. We hypothesised that PFMT and/or CST could reduce subjective UI as compared to no active treatment/standard care.

## Methods

### Study design

A three-armed, two-centred, single-blinded, randomised controlled study was performed. Participants were randomised to one of three groups receiving either PFMT, CST or written information only.

### Subjects

Participants were recruited from physiotherapy departments or pulmonary rehabilitation courses at two hospitals and at local physiotherapy clinics in two separate health regions in Norway. Women aged ≥18 years with clinically stable COPD in GOLD stages I–IV were included [26]. Further inclusion criteria were self-reported UI and the ability to perform a voluntary pelvic floor muscle contraction (VPFMC). Exclusion criteria were unstable COPD, > 4 hospital admissions due to COPD during the last 12 months, neurological conditions, and gynaecological surgery. Patients who had previously been through surgery for incontinence, hysterectomy or other major gynaecological surgery were excluded, as it was considered that surgery might affect the potential reduction of urinary incontinence with conservative treatments.

### Randomisation process

Randomisation was performed using an internet-based, computerised procedure and was stratified on hospital affinity. Clinicians involved in the clinical assessments were blinded to, and had no influence on, the randomisation procedure. Due to the nature of the interventions, participants and physiotherapists were not blind to group allocation.

### Interventions

#### PFMT group

The PFMT programme was based on the principles for increasing PFM strength [27]. Participants received group exercise sessions (1 h) at a local physiotherapy clinic or physiotherapy department at the hospitals once weekly for 16 weeks with guidance from an experienced physiotherapist. The group exercise sessions focused on PFMT, relaxation and breathing techniques. In addition, the participants received a PFMT programme for daily home use [28] and written information about PFMT [29, 30]. Participants were encouraged to perform three sets of 8–12 close to maximum VPFMC daily and to hold the contraction for more than three seconds. Progression of the PFMT included holding the VPFMC for up to ten seconds, adding three fast contractions at the end of the VPFMC, and progressively more challenging starting positions.

#### CST group

Participants attended one group education session (one hour) and 1–2 individual sessions (30–60 min) tailored to individual needs by an experienced cardiorespiratory physiotherapist, as well as receiving CST exercises and written information for home use. Sessions included general information about CST and advice about how to distinguish between unproductive and productive cough to avoid unnecessary coughing.

#### Control group

Participants received instruction on correct VPFMC at clinical assessment (to allow for assessment) and brief written information about PFMT and CST, but no other form of regular follow-up or intervention. PFMT or CST was allowed due to ethical considerations, but not encouraged.

## Materials

### Outcome measures

#### Urinary incontinence

Frequency, volume, and type of UI as well as the overall impact on HRQL was measured using the International Consultation on Incontinence Questionnaire – Urinary Incontinence Short Form (ICIQ-UI SF). ICIQ-UI SF has demonstrated good validity and reliability [31] and is sensitive to change after intervention [32]. Scores range from 0 to 21 points, with a higher score indicating worse severity.

#### Daily physical activity

Daily physical activity and steps per day were monitored for 1 week (24 h per day) before and after the intervention period using a SenseWear armband monitor (BodyMedia Inc., Pittsburgh, PA). SenseWear has been shown to have good reliability, validity and compliance amongst people with COPD [33, 34].

#### Functional exercise capacity

The six-minute walk test (6MWT) was used to measure functional exercise capacity, expressed as the distance walked in 6 min (6MWD). The 6MWT is a valid and reliable outcome measure for assessing exercise tolerance in the COPD population [35, 36]. The test was performed indoors by experienced physiotherapists along a 30-m straight, flat corridor according to guidelines [36]. A practice test was performed to exclude any learning effects.

#### Cough

Physical, psychological and social factors related to cough were measured using the Norwegian version of the Leicester Cough Questionnaire [37, 38]. The questionnaire includes 19 items with a 7-point Likert scale ranging from no symptoms (“Not at all”) to debilitating symptoms (“All the time”). Total scores vary from 3 to 21 points with a higher score indicating less symptoms and better HRQL.

#### Health status

The COPD Assessment Test (CAT) is a disease-specific, reliable and validated questionnaire measuring health status in COPD [39]. Scores range from 0 to 40 points, with a higher score indicating a higher symptom burden.

#### Pelvic floor muscle strength

Participants underwent clinical examination and evaluation of VPFMC function using vaginal palpation and the International Continence Society (ICS) score [40]. The scale ranges from no active muscular contraction (“1”), reduced function (“2”), normal function (“3”) and spastic (“4”). All participants scored ≥2 on the ICS score as according to our inclusion criteria.

#### Lung function measurements

Post-bronchodilator spirometry was conducted on a Master Screen PFT system (CareFusion, Germany). Forced expiratory volume in 1 second (FEV_1_) and forced vital capacity (FVC) were taken as the highest value from at least three satisfactory manoeuvres and FEV_1_/FVC ratio according to Norwegian reference values [41] were used.

In addition, relevant background information such as age, smoking history, parity, mode of child delivery and menopause were collected.

## Procedure

### Statistics

#### Sample size estimation

The primary outcome variable was mean change in ICIQ-UI SF score at post-intervention and a change in mean ICIQ-UI SF score of at least 2 points was considered to be of clinical interest. A power calculation was performed in the hope to perform a full RCT. Based on similar studies and clinical experience, we assumed that a reduction in ICIQ-UI SF scores would be ≥60% within the PFMT group and ≥30% within the CST and control groups due to the placebo effect and being instructed on VPFMC at the initial assessment. Based on results from published studies on UI [3], we assumed that the mean improvement in the ICIQ-UI SF scores in each of the three groups would be 2.5 points. We aimed for a probability of 80% to identify differences between the groups. With a significance level of 5%, the calculation showed that a minimum of 26 participants was required in each group (total of 78 participants).

Descriptive statistics were used to characterise the study population: mean, standard deviation (SD), median, percent and interquartile range as appropriate. The association between ICIQ-UI SF, age, UI duration, VPFMC, functional exercise capacity and health status were examined using Pearson’s product-moment correlation coefficient. Between-group analyses were not performed due to the low number of participants.

Estimated regression coefficients are presented with 95% confidence interval and *p* values.

Statistical significance level was set at 5%. All statistical analyses were performed with SPSS Statistics 26 (SPSS Inc. Chicago, IL, USA).

## Results

### Baseline characteristics

**Fig. 1:**
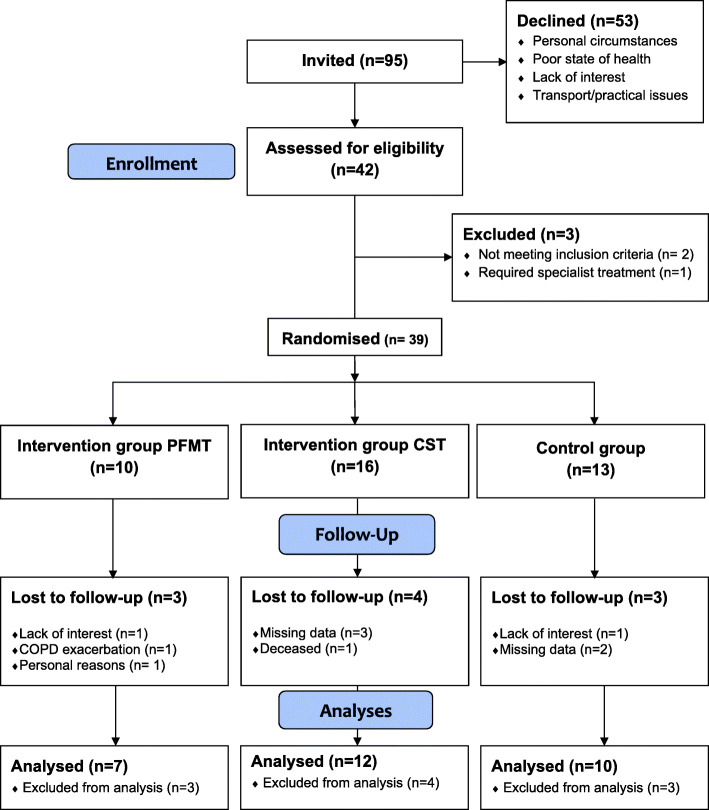
Flow chart of the recruitment and inclusion process through each stage of the randomised controlled trial

Participants were recruited during the period 2016 and 2018. In total, 95 women were invited to participate in the study and 42 women were recruited (Fig. 1). Reasons for women declining to participate are shown in Fig. 1. Three participants were excluded after recruitment and 10 dropped out. Mean age (±SD) was 66.4 (±7.9) years and mean FEV_1_ was 52.7% predicted (±16.8). According to GOLD stages, 15% had mild, 39% moderate, 33% severe and 8% very severe COPD. GOLD stages were missing for two patients (5%) due to a lack of spirometry measures prior to recruitment. Mean ICIQ-UI SF score was 9.6 (±4.3) points suggesting moderate UI severity. VPFMC was weak in 16 women (55%), normal in eight (28%) and spastic in five (17%) at baseline. The overall mean CAT score was 22.1 (±6.8) points indicating a high COPD symptom burden. There were no statistically significant differences at baseline between the completers and non-completers or intervention groups regarding UI severity and duration, VPFMC, physical capacity, lung function, cough symptoms and health status (Table 1) suggesting that these baseline characteristic differences would not affect their outcome. The total scores on the ICIQ-UI SF had a statistically significant correlation with UI duration (*r*=0.59, *p*=0.01), but there was no correlation with the other variables (Table 2). Compliance and dropout were similar in the three groups (Fig. 1). The interventions were well tolerated, and no adverse events were registered.

**Table 1:** Baseline characteristics of study participants (*n*=39)

Baseline	All participants ***n***=39	PFMT group ***n***=7	CST group ***n***=12	Control group ***n***=10	Non-completers ***n***=10
Age (years)	66.4±7.9	67.6±5.5	67.9±5.4	63.9±12.3	66.3±6.9
Current smokers, *n* (%)	12 (31)	1 (14)	4 (33)	5 (50)	2 (20)
Nullipara, *n* (%)	5 (13)	0 (0)	1 (8)	3 (30)	1 (10)
Parity	2.0±1.1	2.3±0.5	2.2±0.9	1.8±1.7	1.8±0.9
UI duration (years)	10.8±11.5	9.7±10.9	9.4±11.9	10.3±11.7	13.2±12.9
FEV_1_ (% pred.)	52.7± 16.8	50.9±12.8	52.0±17.1	57.9±18.9	49.0±18.2
FVC (% pred.)	78.9±14.7	82.7±10.4	75.2±16.5	85.2±16.5	75.1±14.2
GOLD class, *n* (%)					
I	6 (15)	1 (14)	3 (25)	2 (20)	0 (0)
II	15 (39)	2 (29)	2 (17)	5 (50)	6 (60)
III	13 (33)	3 (43)	6 (50)	3 (30)	1 (10)
IV	3 (8)	1 (14)	0 (0)	0 (0)	2 (20)
Missing	2 (5)	0 (0)	1 (8)	0 (0)	1 (10)
6MWD (m)	383±112	382±97	375±160	388±88	387±84
Steps/day	3563±3063	3565±1732	2439±2460	4449±3434	4118±4374
CAT total score	22.1±6.8	24.1±5.7	20.6±6.8	25.5±5.7	18.9±7.2
ICIQ-UI SF total score	9.6±4.3	8.9±4.3	7.3±4.6	11.4±2.8	11.1±4.3
LCQ total median (IQR)	16.0 (14.0–18.8)	15.0 (14.0–20.0)	15.0 (12.8–18.0)	17.0 (13.0–18.2)	18.0 (15.5–20.5)

Data are presented as mean ± SD otherwise stated. *PFMT* pelvic floor muscle training, *CST* cough-suppression therapy, *FEV*_*1*_ forced expiratory volume in 1 second, *FVC* forced vital capacity, *GOLD* Global Initiative for Chronic Obstructive Lung Disease, *6MWD* six-minute walk distance, *CAT* COPD Assessment Test, *ICIQ-UI SF* International Consultation on Incontinence Questionnaire - Urinary Incontinence Short Form, *LCQ* Leicester Cough Questionnaire, *IQR* interquartile range

**Table 2:** The correlation between ICIQ-UI SF and age, duration of UI, pelvic floor muscle contraction, functional exercise capacity and health status at baseline (*n*=29)

Variables	1	2	3	4	5	6
1. ICIQ-UI SF total score	1.00					
2. Age	− .22	1.00				
3. UI duration	.59^**^	.19	1.00			
4. ICS scale	.01	− .17	0.06	1.00		
5. 6MWD	− .18	− .52**	− .20	.17	1.00	
6. CAT	.11	.35	.18	.35	− .38*	1.00

**Correlation is significant at the 0.01 level (2-tailed), *Correlation is significant at the 0.05 level (2-tailed)

*ICIQ-UI SF* International Consultation on Incontinence Questionnaire - Urinary Incontinence Short Form, *UI* urinary incontinence, *ICS scale* International Continence Society scale for evaluation of voluntary pelvic floor muscle contraction, *6MWD* six-minute walk distance, *CAT* COPD Assessment Test

### Changes in UI and activity levels from baseline to post-intervention

**Fig. 2:**
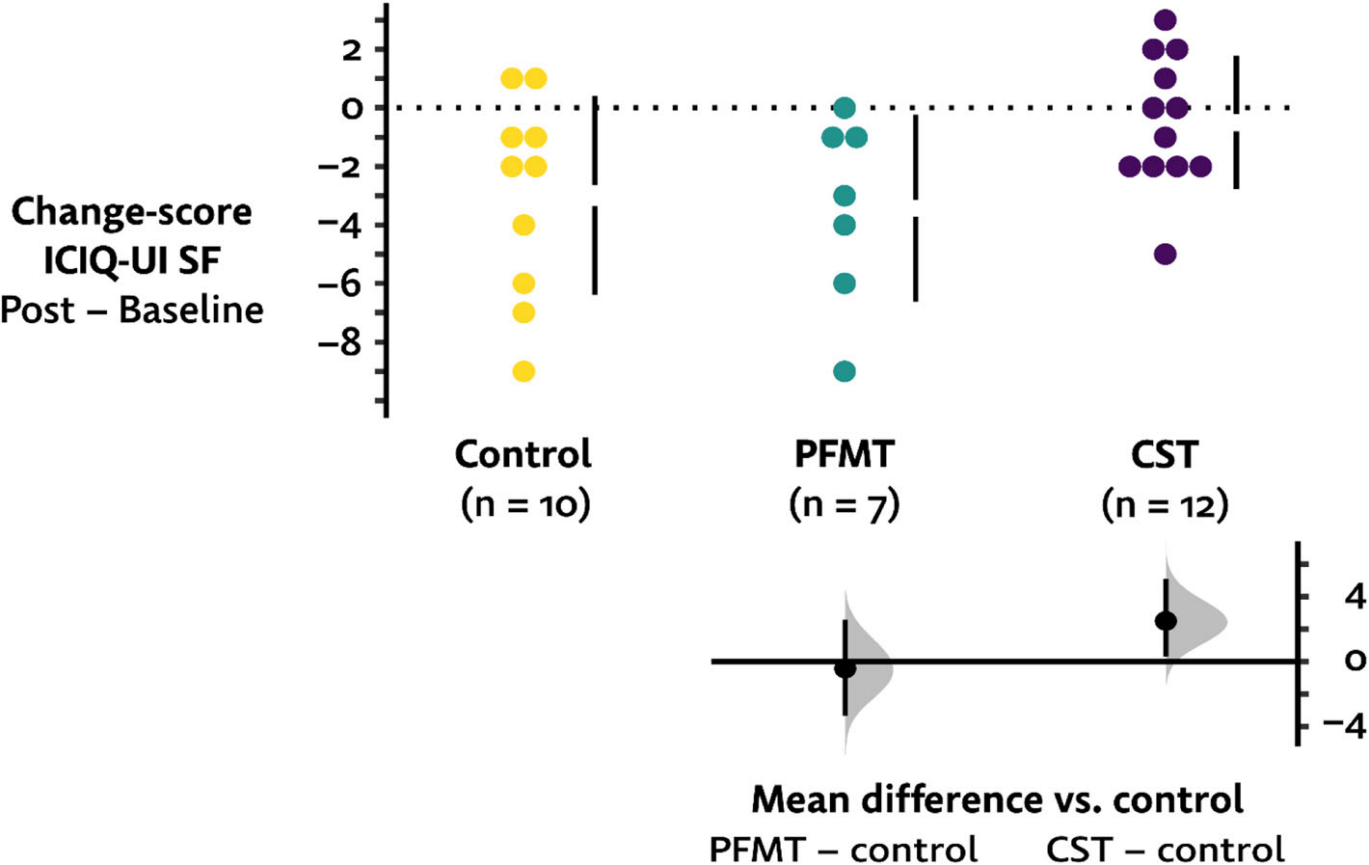
Change in ICIQ-UI SF scores from baseline to post-intervention (*n*=29)

The PFMT group and control group women had a clinically important mean reduction of 3.4 points and 3.0 points, respectively, on the ICIQ-UI SF score (Table 3), whereas the reduction in the CST group was 0.5 points only (Fig. 2). The physical activity level was low in all three groups at baseline (Table 3).

**Table 3:** Mean changes in the intervention and control groups, post- minus pre-test

Variables	Completers ***N***=29	PFMT group ***N***=7	CST group ***N***=12	Control group ***N***=10
**ICIQ-UI total score**				
Baseline	9.1±4.3	8.9±4.3	7.3±4.6	11.4±2.8
Mean change	− 2.1±3.1	− 3.4±3.2	− 0.5±2.3	− 3.0±3.4
Missing	0	0	0	0
**6MWD**				
Baseline	381±121	382±97	375±160	388±88
Mean change	− 7± 0	12±46	− 3±60	− 38±89
Missing	2	0	1	1
**Steps/day**				
Baseline	3416±2762	3565±1732	2439±2460	4449±3434
Mean change	267±1048	218±557	− 93±1001	1053±1514
Missing	16	3	6	7
**CAT total score**				
Baseline	23.1±6.4	24.1±5.7	20.6±6.8	25.5±5.7
Mean change	0.2±6.1	− 2.1±6.3	3.4±4.9	− 1.6±6.3
Missing	1	0	2	0
**LCQ total median**^**1**^				
Baseline	16	15	15	17
Change (median)	0	0	1.0	− 0.5
Missing	2	0	2	0
**ICS baseline**				
Weak, *n* (%)	16	5	7	4
Normal, *n* (%)	8	2	3	3
Spastic, *n* (%)	5	0	2	3
Missing	0	0	0	0
**ICS post-intervention**				
Weak, *n* (%)	7	2	3	2
Normal, *n* (%)	11	5	3	3
Spastic, *n* (%)	4	0	2	2
Missing	7	0	4	3

Data are presented as mean ± SD otherwise stated. *PFMT* pelvic floor muscle training, *CST* cough-suppression therapy, *ICIQ-UI SF* International Consultation on Incontinence Questionnaire - Urinary Incontinence Short Form, *6MWD* six-minute walk distance, *CAT* COPS Assessment Test, *LCQ* Leicester Cough Questionnaire, *ICS* International Continence Society

## Discussion

This randomised controlled study explored the effect of PFMT and CST on treating UI in women with COPD. Due to difficulties with recruitment, such as practical issues regarding travelling distance, lack of interest, poor state of health and high number of comorbidities, we did not reach the number of participants required in each study group as shown in our sample size estimation. Our results demonstrated a trend of reduced UI symptoms amongst women in the PFMT and control groups and no change in the CST group. However, due to insufficient statistical power, the results must be considered with caution.

To our knowledge, this is the first study that has investigated the use of PFMT alone in this patient group. Our findings suggest that PFMT may be an effective treatment for UI in women with COPD. Surprisingly, a reduction in UI symptoms was also observed in the control group. Due to ethical reasons, all recruited women were instructed in how to perform a VPFMC at baseline assessment and were informed of the benefits of PFMT. Therefore, a plausible explanation may be that women in the control group started to perform PFMT on their own initiative following randomisation. Performing PFMT in groups led by a physiotherapist has been found to be more effective than unsupervised home training [42] and therefore one would still have expected a greater improvement amongst the participants who followed a structured programme supervised by a specialised physiotherapist. However, as this is only a small study, larger numbers would be required to explore this further.

UI severity was significantly correlated with UI duration, but we found no other correlations between UI and age, lung function, parity, cough symptoms, VPFMC, 6MWD or health status. The participants were representative for the COPD population in terms of age and disease severity. Despite this, they presented with severe UI symptoms and approximately half had weak VPFMC when measured with vaginal palpation at study inclusion. This was somewhat unexpected as the presumption was that higher grades of UI severity would correlate with COPD severity, chronic cough symptoms, weak PFM, poorer health status and reduced physical activity levels. Studies have previously shown that people with COPD commonly have peripheral muscle weakness as compared to age-matched controls [43] and it is therefore possible that similar muscle atrophy is present in the PFM affecting the ability to perform an effective VPFMC. However, despite the majority of participants having reduced VPFMC at baseline, these women had surprisingly normal PFM strength compared to the general female population with UI, especially when considering their high levels of inactivity and presumed peripheral muscle weakness. VPFMC function was measured in a resting, supine position and not during activities such as walking, jumping, or coughing which place extra pressure on the pelvic floor. Despite the participants being able to contract the PFM in a resting state, it is possible that they are not able to, or forget to, contract the PFM during coughing, sneezing or breathlessness. PFMT aims to improve an automatic co-contraction of the PFM to counteract any increase in abdominal pressure or the increase from ground reaction force, and previous studies suggest that purely teaching patients to contract the PFM during coughing (known as “the knack”) reduces UI by up to 70% [44]. However, our results suggest that the presence of UI in women with COPD is multifactorial. It is possible that these patients adjust their lifestyle to cope with UI or that UI does not affect them negatively compared to other more bothersome symptoms, such as dyspnoea. Despite the mean airflow obstruction representing moderate COPD, study participants reported a high symptom burden on the CAT, suggesting a great deal of challenges related to their COPD. Our findings therefore indicate that UI affects women with COPD regardless of disease severity such as it is probably wise to screen all women with COPD for the presence of UI, irrespective of other factors.

UI has previously been demonstrated to be a barrier for participating in physical activity in general female populations [18]. Therefore, the combination of inactivity in COPD due to dyspnoea and UI may result in a double risk factor for reduced compliance to physical activity. Despite the study participants being generally very inactive, the SenseWear activity monitor did not demonstrate a correlation between daily physical activity and any other variables including UI severity and lung function. However, due to the low response rate, and a lack of case-controls, it is difficult to conclude whether this convenience sample of COPD women with coexisting UI is representative for the population. Our participants may not represent women with mild or severe COPD and UI as they are less likely to attend pulmonary rehabilitation or physiotherapy clinics and are thus not available for recruitment.

During the three-year study period, a number of measures were attempted to recruit participants, such as visiting COPD exercise groups to inform patients and clinicians about the study, advertising via social media, and making telephone calls to potential participants to encourage them to participate. In addition, the recruiting phase of the study was extended and a second study centre in a separate Norwegian health region was included in the study. However, it was difficult to engage patients despite many of them reporting problems with UI. Despite a high number of patients in both health regions were invited to the study, our study did not reach the required sample size. The involvement of patients in the design stage of the study may have provided valuable information about relevant research questions from the patients’ perspective, as well as potentially affecting the choice of outcome assessments. This may in turn have resulted in fewer difficulties with recruitment and resulted in clinical relevant research questions.

The assessment of VPFMC included an internal examination (vaginal palpation) which may have deterred potential participants from being recruited to the study, due to embarrassment or discomfort. Despite this being a potential barrier to recruitment, assessment of the participants’ ability to perform VPFMC prior to PFMT is advised, as it is unlikely that women who are unable to perform VPFMC at baseline will benefit fully from PFMT alone. VPFMC was therefore considered an important outcome measure directly addressing the research in this study. Women unable to contract their PFM at baseline may potentially require supplemental treatment, such as electrical stimulation, in order to improve their PFM strength and reduce UI symptoms had they been allocated to the PFMT intervention group [4].

### Limitations

Due to the small sample size and relatively high dropout rate, it is difficult to conclude the effectiveness of the interventions, as previously discussed. Furthermore, CST is not a well-documented or standardised treatment, and this may have resulted in less than optimal results in the CST group. Future qualitative studies to identify the barriers to recruitment in this patient group, such as practical issues with transport, beliefs about the effect of treatment, and reducing embarrassment about the somewhat taboo issue of UI, may provide an insight on how UI affects women with COPD, including those unable to participate in a research study. Studies should also include women with milder COPD and UI where the potential to prevent the development of UI may be greater. Despite this, our results may still lead to increased awareness regarding UI in patients with COPD, as well as giving an indication to which interventions may be effective. This in turn may result in better patient care by extending the knowledge base for prevention, diagnostics, and treatment of UI amongst patients with COPD. Additionally, further research is required to explore the relationship between VPFMC and general strength in this population.

## Conclusion

Due to the low number of available participants and recruitment difficulties, including practical issues such as travel distance, lack of interest, poor state of health and high number of comorbidities, our results are inconclusive. However, our results support previous evidence that PFMT is the primary choice of treatment for UI in the general female population, as our findings suggest that PFMT may be an effective treatment for women with COPD and coexisting UI. Increased knowledge about COPD and coexisting UI may lead to improved routines for the assessment, prevention and treatment of UI amongst this population, as well as increasing awareness and reducing taboos around the subject of UI amongst COPD patients. Routine screening for symptoms of UI amongst people with UI may provide the opportunity to identify, educate and treat these patients, which may lead to reduced symptom burden and improved quality of life. More research exploring the association between COPD and coexisting UI is needed, as well as larger studies investigating barriers to recruitment and treatment options for reducing UI amongst COPD patients.

## Data Availability

The datasets used and/or analysed during the current study are available from the corresponding author on reasonable request.

## Abbreviations

UI: Urinary incontinence
COPD: Chronic obstructive pulmonary disease
PFMT: Pelvic floor muscle training
CST: Cough-suppression therapy
PFM: Pelvic floor muscles
HRQL: Health-related quality of life
SUI: Stress urinary incontinence
GOLD: Global Initiative for Chronic Obstructive Lung Disease
FEV_1_: Forced expiratory volume in 1 second
FVC: Forced vital capacity
VPFMC: Voluntary pelvic floor muscle contraction
6MWT: Six-minute walk test
LCQ: Leicester cough questionnaire
CAT: COPD Assessment Test
ICS: International Continence Society score
RCT: Randomised controlled trial
SD: Standard deviation
6MWD: Six-minute walk distance
PROMS: Patient-reported outcome measures
